# Long COVID: The latest manifestations, mechanisms, and potential therapeutic interventions

**DOI:** 10.1002/mco2.196

**Published:** 2022-12-08

**Authors:** Shi‐ting He, Kexin Wu, Zixuan Cheng, Mengjie He, Ruolan Hu, Ning Fan, Lin Shen, Qirui Li, Huahao Fan, Yigang Tong

**Affiliations:** ^1^ College of Life Science and Technology Beijing University of Chemical Technology Beijing China

**Keywords:** breakthrough infection, long COVID, SARS‐CoV‐2, SARS‐CoV‐2 variants

## Abstract

COVID‐19 caused by SARS‐CoV‐2 infection affects humans not only during the acute phase of the infection, but also several weeks to 2 years after the recovery. SARS‐CoV‐2 infects a variety of cells in the human body, including lung cells, intestinal cells, vascular endothelial cells, olfactory epithelial cells, etc. The damages caused by the infections of these cells and enduring immune response are the basis of long COVID. Notably, the changes in gene expression caused by viral infection can also indirectly contribute to long COVID. We summarized the occurrences of both common and uncommon long COVID, including damages to lung and respiratory system, olfactory and taste deficiency, damages to myocardial, renal, muscle, and enduring inflammation. Moreover, we provided potential treatments for long COVID symptoms manifested in different organs and systems, which were based on the pathogenesis and the associations between symptoms in different organs. Importantly, we compared the differences in symptoms and frequency of long COVID caused by breakthrough infection after vaccination and infection with different variants of concern, in order to provide a comprehensive understanding of the characteristics of long COVID and propose improvement for tackling COVID‐19.

## Graphical abstract

Long COVID manifests in different organs of different patients. In this manuscript, symptoms, mechanism, and potential interventions of long COVID in different organs were discussed as follows: pulmonary and respiratory involvement, neurological involvement, smell and taste dysfunction, vascular involvement, muscular involvement, cardiac involvement, intestinal involvement, immunological dysfunction, kidney involvement, biochemical abnormalities of liver, ocular involvement, and sexual dysfunction. In addition, the differences in symptoms and frequency of long COVID caused by breakthrough infection after vaccination and infection with different variants of concern are discussed in the manuscript.

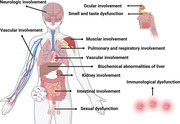

## 1 INTRODUCTION

The pandemic caused by SARS‐CoV‐2 infection has been lasted for two and a half years, billions of people have been infected and recovered. In the early period of the epidemic, most patients manifested multiple organs symptoms, mainly pulmonary symptoms, while the emerging variants omicron infection caused mild symptoms and the proportion of hospitalized cases is significantly reduced. However, similarly to SARS‐CoV and MERS‐CoV infections, some patients recovered from SARS‐CoV‐2 present residual symptoms after the resolution of acute viral infection. The World Health Organization (WHO) recommends a maximum recovery time of 2 weeks for mild disease and 6 weeks for severe disease.^1^ Therefore, post‐infectious symptoms longer than 6 weeks can be considered as long COVID. The SARS‐CoV‐2 variant omicron has developed its dominance throughout the world with enhanced transmissibility.^2^, ^3^ It is necessary to elucidate the mechanism of long COVID and develop the guidance for management of post‐COVID‐19 infection to cope with the challenge that omicron epidemic brings.

In this review, we illustrated the prevalence and manifestation of multiple long‐term symptoms caused by COVID‐19 based on case reports and longitudinal studies of COVID‐19 survivors at various stages of the epidemic, and elucidated the potential mechanisms based on in vitro and in vivo studies. According to the manifestation and mechanisms, we provided management suggestions on different long COVID symptoms. The long COVID symptoms were reviewed in the order of their general prevalence at COVID‐19 convalescence. Moreover, we firstly reviewed the long COVID in patients with breakthrough infection, and compared the discrepancy of long COVID in patients infected with different SARS‐CoV‐2 variants. The lower tropism of omicron to lungs and olfactory epithelium may decrease the incidence of pulmonary involvement and smell/taste loss. The less replication efficiency of omicron in olfactory epithelium may reduce neurotropism, neuro‐invasion, and neurovirulence compared to earlier strains. As SARS‐CoV‐2 vaccination becomes prevailing and the virus continues to evolve, our understanding of long COVID needs to be continuously updated to provide timely information for public policy and guidance for post‐COVID‐19 care.

## 2 MANIFESTATIONS OF LONG COVID IN MULTIPLE ORGANS

### 2.1 Pulmonary and respiratory involvement

SARS‐COV‐2 is a respiratory virus, and fever, cough, and dyspnea are the most common symptoms of COVID‐19 patients before the epidemic of omicron. COVID‐19 is a multisystem disease, and the lung is the main target of infection and injury. In severe cases, it can lead to acute respiratory distress syndrome.^4^ One of the typical chronic symptoms in early COVID‐19 convalescent is respiratory damage.^5^, ^6^, ^7^, ^8^, ^9^, ^10^ A study based on patients during the early epidemic showed the prevalence of dyspnea in COVID‐19 patients was 30% at 1.5 months post‐infection, and was still over 9% at 7 months after recovery.^4^ A 2‐year follow‐up study of hospitalized patients in Wuhan showed 26% of patients experienced dyspnea at 6 months post‐infection, and significantly reduced to 14% at 2 years post‐infection.^11^ Half of COVID‐19 patients had experienced dyspnea at 3 months post‐infection, and females under 50 years old were more likely to suffer from this.^12^ Therefore, dyspnea is the main long COVID symptom of the lung and respiratory system in the early epidemic, which increased the demand for oxygen after recovery,^13^ decreased the vital capacity, and impaired the cardiopulmonary function and exercise ability.

Persistent respiratory symptoms in COVID‐19 patients may be related to the development of pulmonary fibrosis. The follow‐up computed tomography scan of severe COVID‐19 patients at convalescence revealed the fibrotic interstitial lung abnormalities, which leads to the reduction of lung carbon monoxide diffusion capacity,^14^ which is mostly used in clinical to evaluate the gas exchange ability of lungs. Substantial studies had shown that COVID‐19 patients had decreased lung diffusion capacity.^6^, ^15^, ^16^, ^17^ A 1‐year follow‐up of the COVID‐19 convalescents showed that nearly 25% of them had persistent imaging abnormalities with fibrosis characteristics. The incidence of these long‐term abnormalities was positively correlated with disease severity.^4^ Dyspnea and cough were seen remission at 2 months post‐infection in mild to moderate COVID‐19 convalescents,^5^, ^9^, ^18^, ^19^, ^20^ and the pulmonary fibrosis at 1 year post‐infection was rarely detected, and the lung function and exercise capacity were within the normal range,^21^ indicating the risk of pulmonary long COVID in mild COVID‐19 patients is negligible.^22^ In addition to the direct infection of the virus in lungs, severe cytokine storm damages normal tissues and organs and causes lung injury, resulting in dyspnea and other symptoms. The severe long‐term systemic inflammation contributed to persistent abnormalities in the structure and function of lungs.^23^

### 2.2 Neurological involvement

COVID‐19 causes a series of neurological involvements, mainly including fatigue, headache, sleep disturbances, and smell and taste loss.^24^, ^25^ Manifestations of neurological sequelae in COVID‐19 varied with the time of discharge. Female was more likely to experience general long COVID neurological symptoms.^26^ Fatigue was the most common post‐acute symptom for COVID‐19 in both inpatients and outpatients lasting for several months, while inpatients were associated with higher risk of general neurological sequelae than outpatients.^27^, ^28^ In the early epidemic, the prevalence of fatigue in 1 year post‐infection was 50%–70%.^28^, ^29^, ^30^ The female, severe COVID‐19 patients, the elderly, and those with headache in the acute phase had higher odds of having fatigue.^25^, ^28^, ^30^, ^31^ Headache occurred in 16.0%–19.0% of patients 3–9 months after infection.^32^ Post‐COVID headache adopted two phenotypes, tension‐type‐like and migraine‐like headache, with short‐term long COVID headache mainly manifested as migraine‐like.^33^, ^34^ Female, people with past migraine history or headache at the time of onset, and the elderly are more likely to develop long‐term post‐COVID headache.^32^, ^33^ Similar to the causes of fatigue, the development of headache at onset or in convalescence was facilitated by the presence of a prolonged pro‐inflammatory response, which led to overexcitability of the renin–angiotensin (Ang) system.^33^

The prevalence of post‐traumatic stress disorder (PTSD) was significantly higher in COVID‐19 patients than in normal population,^35^ and decreased during 3 months follow‐up.^36^ Unlike anxiety and depression, a lower prevalence of long COVID PTSD was reported in female,^37^ and degree of PTSD was independent of the severity of COVID‐19.^36^, ^38^

Studies showed that long COVID depression and anxiety improved over time.^36^, ^39^ Females showed lower levels of inflammatory markers, but were more susceptible to anxiety and depression.^38^ The severity of COVID‐19 and virus‐induced inflammation positively contributed to the long COVID depression and anxiety.^36^, ^38^, ^40^ In addition, comorbidities also played crucial roles in the development of depression and anxiety. Previous psychiatric history was considered to be the predictor of depression and anxiety.^39^ Obsessive‐compulsive disorder (OCD) is a type of anxiety disorder, which is characterized by the co‐existence of conscious compulsion and anti‐compulsion. A study found that 20% of patients in early COVID‐19 epidemic suffered from OCD at 1 month follow‐up,^38^ and signs of OCD improved in 1–3 months follow‐up.^36^ Females had a higher risk of OCD.^41^

COVID‐19 convalescents were more likely to experience cognitive impairment than normal individuals, persisting for 2–12 months,^36^, ^42^, ^43^ and improved over time.^44^ Cognitive impairment is manifested as impaired verbal memory, learning ability, attention, working memory, and language fluency.^42^, ^45^ The occurrence of cognitive impairment was associated with the severity of the infection and systemic inflammation.^36^

Symptoms including inattention, amnesia, blurred consciousness, depression, and fatigue were collectively referred to “brain fog.” A study in 2020 showed that 29% of COVID‐19 convalescents reported the presence of “brain fog” in 6 months post‐infection.^46^

Some studies suggested that smell and taste loss belonged to neurological involvement, while others showed that olfactory neuron lackedthe expression of angiotensin converting enzyme 2 (ACE2)nd transmembrane protease serine 2 (TMPRSS2), and transient smell dysfunction was not caused by neuronal destruction, which may be mediated by transient dysfunction of the olfactory epithelium. Smell and taste loss are discussed separately due to the specificity and prevalence in COVID‐19 convalescents.

**FIGURE 1:**
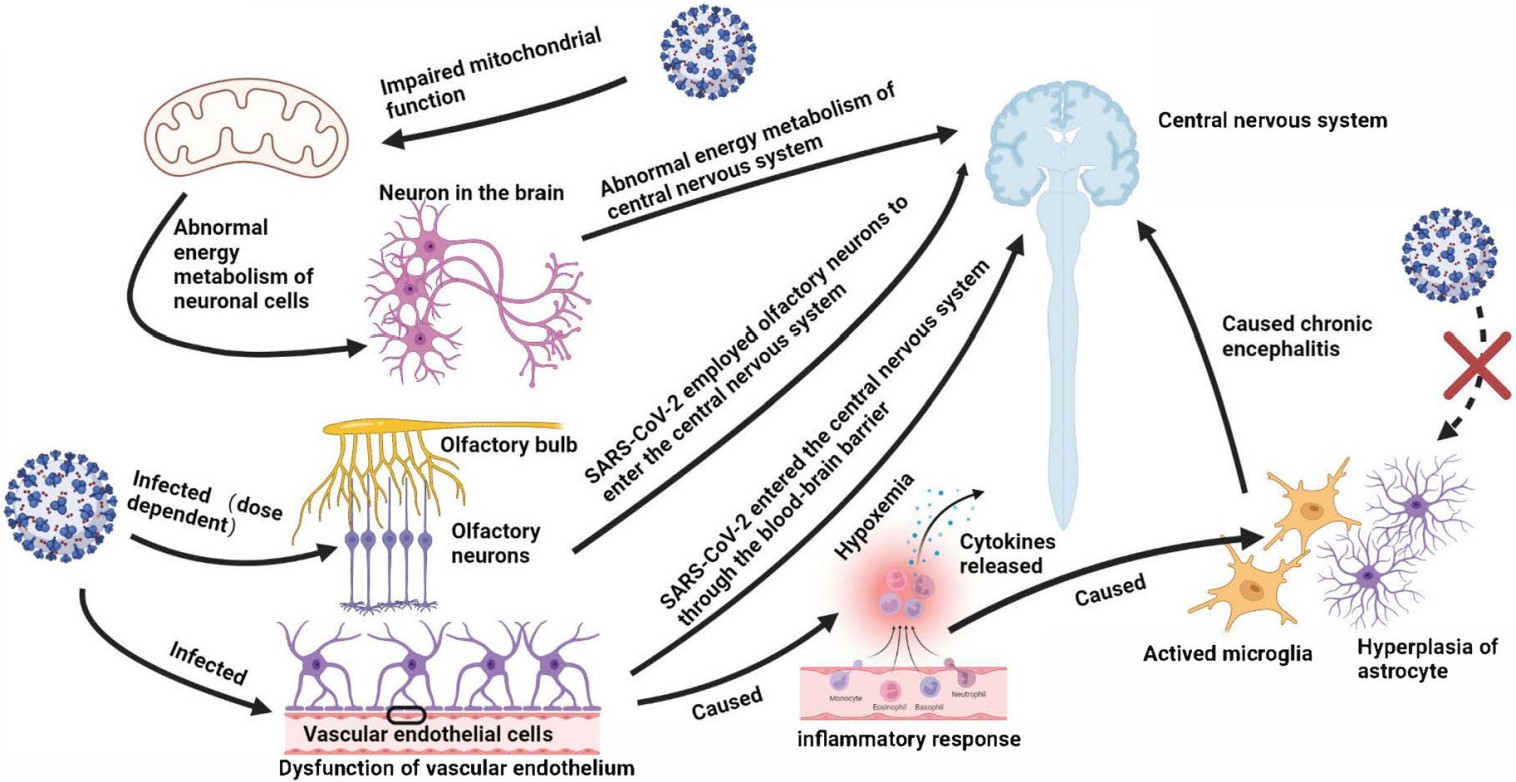
Putative mechanisms of neurological involvement. (1) SARS‐CoV‐2 employs olfactory neurons to enter the central nervous system. (2) SARS‐CoV‐2 enters the central nervous system through the blood–brain barrier. (3) SARS‐CoV‐2 infection induces mitochondrial dysfunction, leading to the abnormal energy metabolism of neurons. (4) SARS‐CoV‐2 infection induces cytokines release

Putative mechanisms of neurological dysfunction in COVID‐19 mainly include direct neural invasion, neurotoxicity caused by inflammatory response and cytokines release, and hypoxemia (Figure 1). The viral protein and RNA of SARS‐CoV‐2 were detected scattered throughout the brain tissues of died and survived patients, and infected K18‐hACE2 mice.^47^, ^48^ Moreover, SARS‐CoV‐2 may employ olfactory neurons or other bundles to enter the central nervous system from the peripheral nervous system or through the blood–brain barrier.^49^, ^50^ Alterations in immune system might be responsible for fatigue, as SARS‐CoV‐2 could reach the hypothalamus via the olfactory pathway, which led to the production of pro‐inflammatory cytokines, and then caused headache and fatigue.^25^ SARS‐CoV‐2 was detected to infect brain vascular endothelial cells and cause brain damage in K18‐hACE2 transgenic mice and hamster model, indicating that SARS‐CoV‐2 crossed the blood–brain barrier to the brain, and led to neuronal damage and inflammatory response. The activation of microglia and astrogliosis led to chronic encephalitis and ultimately the long‐term neurological dysfunction.^26^, ^47^, ^51^, ^52^ In addition, studies have established the relationship between fatigue and mitochondrial dysfunction, and oxidative stress. The normal function of central nervous system demands high levels of adenosine triphosphate produced (ATP) by mitochondria. The mitochondria of infected cells are hijacked for virus replication and assembly, which alters the normal intracellular redox state and leads to oxidative stress. SARS‐CoV‐2 infection was identified to induce brain microstructure changes months after recovery, which injury was related to emotional and sleep disorders,^53^, ^54^ and could also cause migraine, and smell/taste loss.^55^ Nevertheless, studies found that SARS‐CoV‐2 demonstrated its viral tropism for choroid plexus epithelial cells rather than neurons or glial cells,^56^ and biomarkers of central nervous system recovered to normal at convalescence, suggesting the neurological sequelae were not accompanied by neurodegeneration and astrocytic injury.^46^ The cerebrospinal fluid samples from COVID‐19 patients with severe neurological symptoms were generally tested for SARS‐CoV‐2 RNA negative.^47^, ^57^ The above observations suggest that neurological symptoms are unlikely to be driven by direct neural invasion. Certain genetic variants of antioxidant enzymes, including GST family and NRF2, were identified to affect the development of long COVID neurological sequelae. The mitochondrial dysfunction caused by SARS‐CoV‐2 infection, leading to decreased metabolic activity of the central nervous system, may be responsible for the dysregulated immune response and fatigue.^58^ Oxidative stress and mitochondrial dysfunction are believed to involve in the development of chronic fatigue after SARS‐CoV‐2 infection.

### 2.3 Smell and taste dysfunction

Smell and taste loss are specific symptoms of COVID‐19,^59^ and taste loss is based on the loss of smell.^60^ Taste depends on the activity of specialized epithelial cells, which are mainly present in the tongue mucosa.^61^ In addition to high fever and persistent cough symptoms, loss of smell and taste are potential predictors of COVID‐19 before the epidemic of omicron. In the early epidemic, the incidence of smell and taste loss in COVID‐19 individuals was 66%–68%, indicating that taste and smell loss were widespread in COVID‐19 patients and were the strongest predictors of coronavirus infection.^62^ Olfactory and gustatory dysfunction are the most common sensory defects in COVID‐19 patients.^63^ In most hospitalized cases, recovery of olfactory function was rapid (about 10 days), but the residual and insignificant impairment and perceptual distortion might remain for weeks to months.^64^, ^65^, ^66^ Even 46% of mild COVID‐19 convalescents still had olfactory dysfunction more than 1 year after infection, and 7% of them suffered from smell loss.^67^ Compared with the early strains, the risk of chemical sensory loss of alpha, delta, and omicron variants was gradually decreasing (from 50% to 17%).^68^

Females are more likely to report smell or taste loss than males.^59^, ^69^ Female mice presented higher risk of olfactory loss than male mice,^64^ which corresponded to the higher population of smell or taste loss in female patients. The infection results of K18‐hACE2 transgenic mice showed that the virus might enter the central nervous system through other pathways.^64^

**FIGURE 2:**
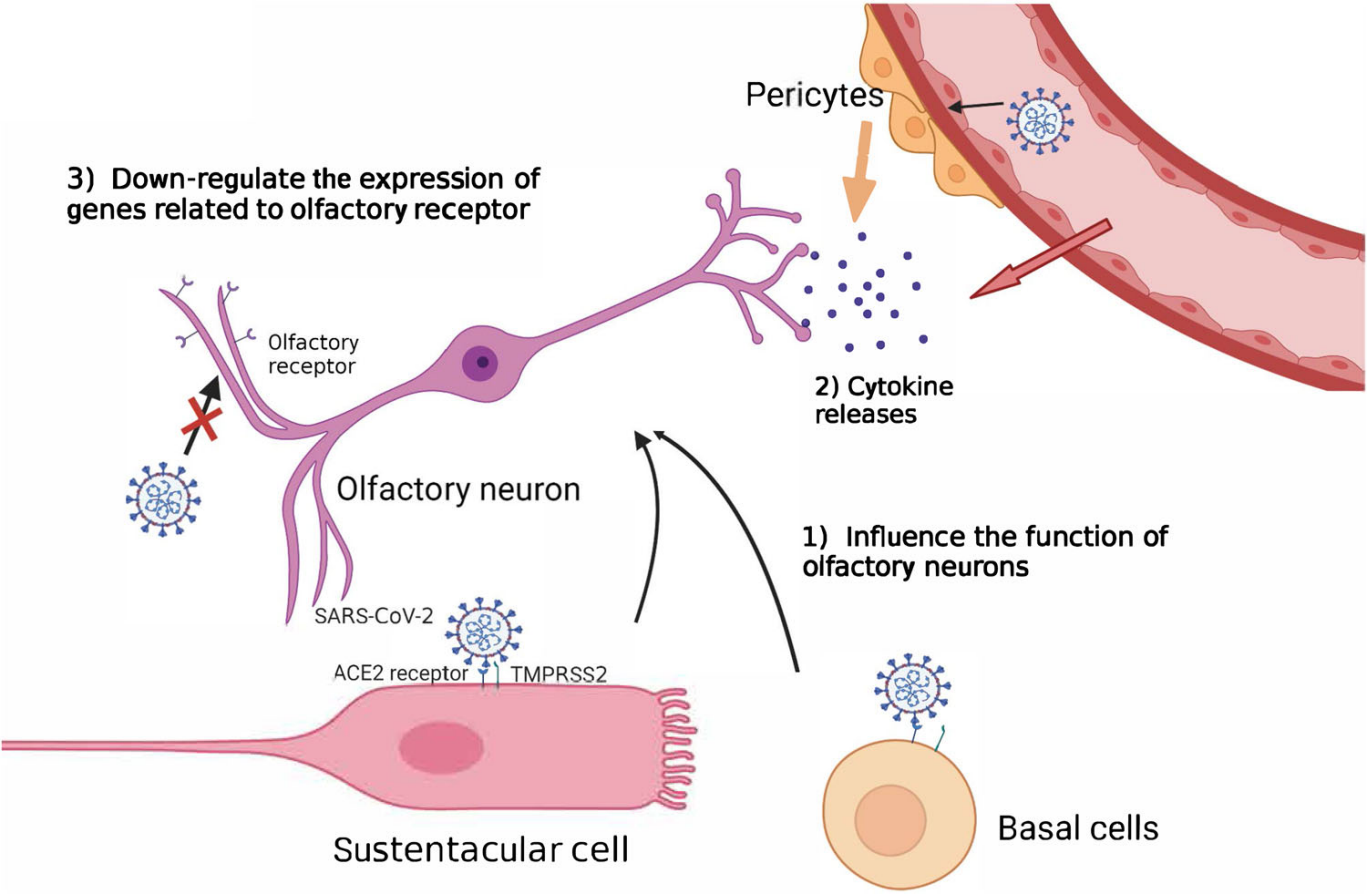
Putative mechanisms of olfactory impairment. (1) SARS‐CoV‐2 infects sustentacular cells and basal cells, which structurally support olfactory neurons and their function impairment leads to the dysfunction of olfactory neurons. (2) SARS‐CoV‐2 infection induces cytokines release, and therefore affects the activity of olfactory neurons. (3) SARS‐CoV‐2 infection down‐regulates the expression of genes related to olfactory receptors

Olfactory deficiency accompanied by rhinorrhea and nasal congestion is normal in respiratory infections because rhinorrhea and nasal congestion isolate olfactory sensory neurons from odorants.^63^ However, olfactory impairment and loss in COVID‐19 infection may attributed to neurological abnormalities. Studies have suggested that olfactory impairment belonged to neurological impairment. The early evidence revealed that olfactory damage caused by infection was caused by indirect damage, rather than the direct infection of sensory neurons.^64^, ^70^ Sustentacular and basal cells from olfactory epithelium infected with SARS‐CoV‐2 may cause olfactory impairment through multiple mechanisms^70^ (Figure 2). SARS‐CoV‐2 spreads through the olfactory epithelium, vascular cells, and pericytes in the olfactory bulb, and impairs signal transduction from olfactory neurons to the brain, which indirectly triggers the death of olfactory neurons.^70^ However, the viral infection of sustentacular cells is a transient event, while some patients recovered from COVID‐19 exhibited long‐term olfactory and gustatory disorders.^63^ Therefore, the viral infection of sustentacular cells may not perfectly interpret the long‐term deficiency of olfactory caused by SARS‐CoV‐2. It was found that the neurons of the deceased who died of COVID‐19 were intact, but the nucleuses of neurons were damaged, and the number of olfactory receptors anchoring on their cell membranes was lower than normal value.^63^ It is speculated that SARS‐CoV‐2 infection down‐regulated the expression of genes related to olfactory receptor. UGT2A1 and UGT2A2, which express in olfactory epithelium have significant mutation, and are involved in the metabolism of odor molecules, was identified in COVID‐19 patients with smell and taste impairment, increasing the possibility of patients losing their smell or taste by 11%^59^ (Figure 2). This finding may explain the long‐term olfactory loss suffered by some COVID‐19 patients.

### 2.4 Vascular involvement

SARS‐CoV‐2 is also found in the cardiovascular (CV) system and other tissues. Therefore, CV injury is one of the causes of a large number of COVID‐19 deaths.^71^ Li et al. performed proteomic analysis on 300 COVID‐19 patients and found alterations in coagulation pathways and the endothelium/vasculature system. They proposed the cascade reaction between vascular and tissue damage and SARS‐CoV‐2 plasma viremia.^72^ Symptoms including hypercoagulability, pulmonary intravascular coagulation, microvascular lesions, venous thromboembolism (VTE) or arterial thrombosis, and endothelial dysfunction were also found in crucial clinical observations of some COVID‐19 patients.^73^, ^74^ All these indicated that the CV system was involved in the symptoms of COVID‐19. It was found that some COVID‐19 convalescents still had CV magnetic resonance abnormalities, VTE, and other vascular abnormalities,^75^, ^76^, ^77^ which suggested vascular involvement as a phenotype of long COVID. Ongoing endothelial dysfunction that caused by sustained activation of endothelial cells was found in COVID‐19 convalescents, and improved during 6 months post‐infection, but it was still impaired compared with the healthy individuals.^78^, ^79^

The direct viral infection on perivascular cardiomyocytes and pericytes, and endothelial dysfunction were mainly responsible for vascular dysfunction in COVID‐19 patients.^73^ ACE2 is vital to the CV and immune systems. SARS‐CoV‐2 infection reduced the levels of membrane ACE2 expression. ACE2 counter‐regulated the renin–angiotensin–aldosterone system (RAAS), which may enhance vasoconstriction. The dysfunction of RAAS and Kinin–Kallikrein System, the opposing regulation to the RAAS, are attributed to CV complications and sequalae. In surviving COVID‐19 patients, multiple fenestrae with comparable diameter of the SARS‐CoV‐2 nucleocapsid were observed on the membrane of endothelial cells, which might be caused by replicated penetration of SARS‐CoV‐2. The damage increased the permeability of cells, leading to tissue edema, inflammation, platelet activation, and enhanced thrombosis.^80^ In addition, endothelial dysfunction was associated with pulmonary complications and other organic damages.^81^ The highly pro‐inflammatory cytokine response induced by SARS‐CoV‐2 infection can cause endothelial dysfunction.^82^ For example, cytokines such as tumor necrosis factor (TNF)‐α and interleukin (IL)‐1β had pro‐inflammatory effects on the endothelium and might involve in the vascular dysfunction of COVID‐19,^78^, ^82^ leading to the coagulopathy.^83^

Persistent endothelial cell dysfunction for more than 2 months is independent of the acute phase reaction or the formation of neutrophil extracellular traps, and related to the enhanced production of thrombin.^78^ Thrombin may play a role in the sustained activation of endothelial cells.^78^ Endothelial glycocalyx (eGC) covering the luminal surface of endothelial cells helps to maintain vascular homeostasis, while obvious eGC disruption was observed in the early stage of critical patients, which may also be a potential key marker of later extensive endothelial injury in severe COVID‐19.^84^

### 2.5 Muscular involvement

Musculoskeletal symptom is one of the most frequent manifestations during the acute phase of COVID‐19.^85^ Muscle damage was one of the sequelae of COVID‐19, and it was mainly manifested as myalgia, arthralgia, muscle weakness, skeletal muscle pain, and decreased hand grip strength. A meta‐analysis has shown that by mid‐2021, nearly 10% of COVID‐19 patients were present with musculoskeletal pain during 1 year after the infection.^86^ Muscle pain was the most visual manifestation of the sequela in muscle injury, with 10%–20% of patients presenting with muscle pain, 20%–40% with joint pain, and 20%–50% with muscle weakness.^29^, ^85^, ^86^, ^87^ Research at 6 months post‐infection showed that around 15% of convalescents still exhibited muscle weakness, and obese and severe patients were at higher risk of developing muscle weakness sequelae 6 months after infection.^87^ Hand grip strength test, a simple method to judge myasthenia gravis, showed a significant decrease in grip strength after COVID‐19, indicating the damage to skeletal muscles. The more pronounced decrease in grip strength was observed in moderate to severe patients.^34^, ^88^ Skeletal muscle mass decreased significantly in severe patients, and more than half of those with pre‐existing musculoskeletal pain presented with worsening symptoms after discharge. Female, people with a history of musculoskeletal pain or myalgia and headache as a symptom at hospital admission with SARS‐CoV‐2 infection were associated with the long COVID musculoskeletal pain.^34^, ^86^, ^89^, ^90^

**FIGURE 3:**
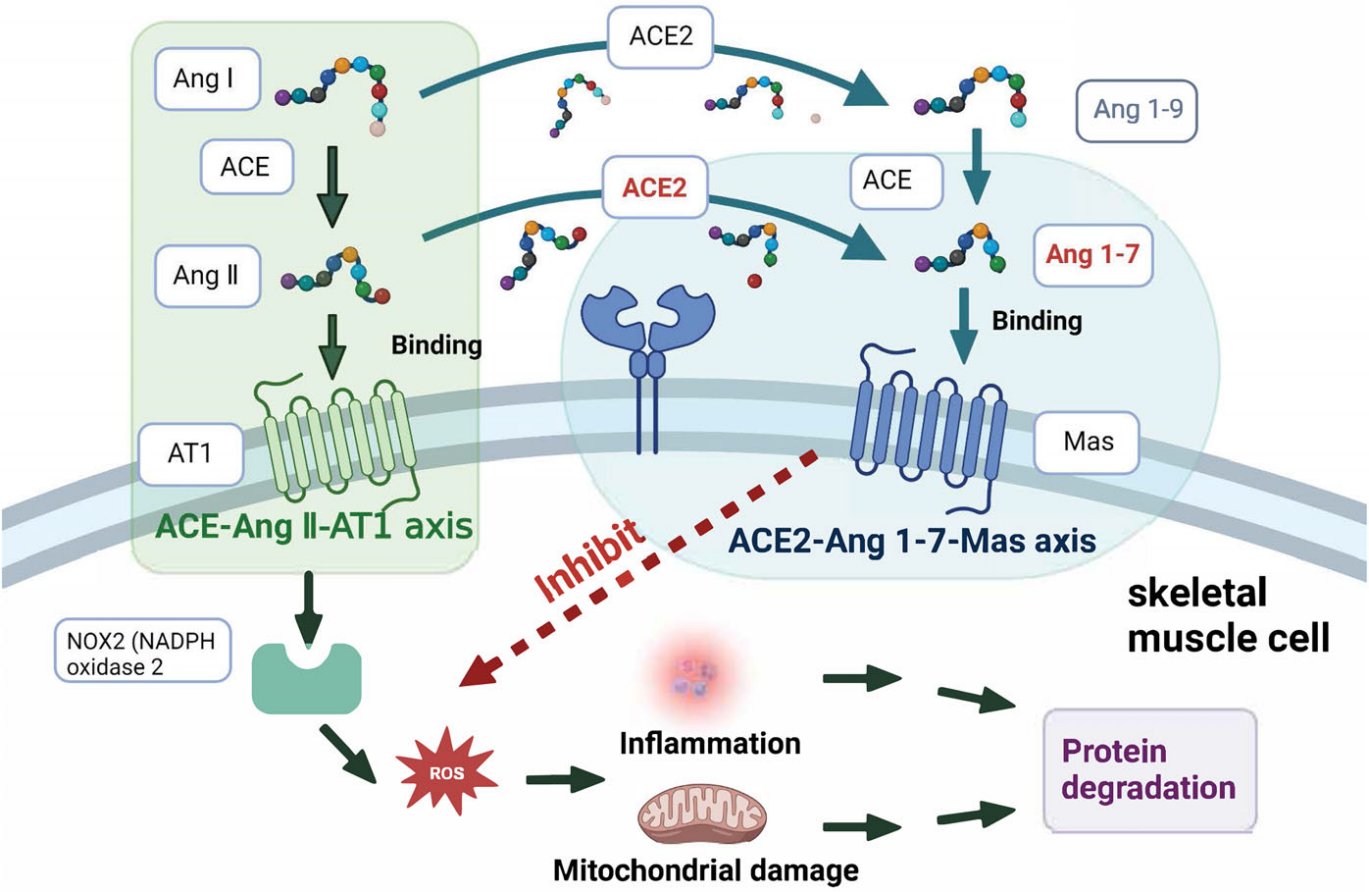
The counter regulation of ACE2–Ang 1–7–Mas axis to ACE–Ang II–AT1 axis, which involve in the protection of muscle wasting. SARS‐CoV‐2 infection down‐regulates the level of ACE2 in the outer‐membrane, and reduces the transformation of Ang 1–7 peptide from Ang II. The increased level of Ang II accelerates the reactive oxygen species (ROS) production through nicotinamide adenine dinucleotide phosphate (NADPH) oxidase 2, which leads to protein degradation and muscle fibrosis. Ang 1–7 combining with Mas is able to inhibit protein degradation induced by NADPH oxidase 2

Single‐cell RNA sequencing and bulk RNA sequencing data suggested that SARS‐CoV‐2 might directly infect skeletal muscle, synovium, and cortical bone, producing direct damage to muscle cells or the surrounding vasculature, with damage extending to skeletal muscle cells, resulting in decreased muscle strength.^34^, ^87^, ^88^, ^91^ However, SARS‐CoV‐2 has not been detected and isolated in these tissues.^92^ Systemic inflammation was also a potential factor in muscular involvement during and after COVID‐19. Increase levels of pro‐inflammatory mediators led to multi‐organ damage, including the muscle.^93^ Pro‐inflammatory mediators have been reported to induce proteolysis in muscle fiber and intervene the process of muscle fiber growth.^91^ The ACE2–Ang (angiotensin) 1–7–Mas axis of skeletal muscle plays a role in preventing the development of insulin resistance or muscle wasting, which is a major pathway to counteract the ACE (angiotensin converting enzyme)–Ang II–AT1 (angiotensin type 1 receptor) axis (Figure 3). Muscle fibrosis is characterized by the replacement of muscle fibers by connective tissue. Ang II plays a pivotal role in inducing muscle fibrosis by activating nicotinamide adenine dinucleotide phosphate (NADPH) oxidase 2.^94^ ACE2 is a major enzyme in producing Ang 1–7 in the tissue. The role of Ang 1–7 in muscle disorders has been extensively reviewed previously, which mediates multiple intracellular signaling pathways including inhibiting reactive oxygen species (ROS) production by NADPH oxidase, and prevents muscular fibrosis and inflammation. Notably, a study found that muscle weakness was independently associated with partial pressure of oxygen in venous blood (PvO2).^95^ Abnormal peripheral oxygen uptake leads to lower PvO2, and muscle hypoxia disrupts the dynamic balance of protein synthesis and degradation in skeletal muscle, which leads to the decrease of muscle mass and does not provide sufficient energy for the body. In addition, muscle hypoxia activates crosstalk in key signaling pathways of oxidative stress and inflammation, leading to disturbances in lipid and glycol‐metabolism, which manifest as muscle atrophy and muscle weakness.^34^, ^95^

### 2.6 Cardiac involvement

Myocardial injury is a prevalent complication and sequelae in early COVID‐19 patients,^96^ which can be determined by electrocardiograph, cardiac magnetic resonance, and biomarker testing.^96^ Viral infection is one of the important causes of myocarditis.^97^ Nearly half of COVID‐19 hospitalized patients before the first half of 2020 were diagnosed with myocardial injury.^98^ Persistent myocardial injury after COVID‐19 infection is manifested by accumulation of inflammatory cells in the myocardium,^99^ continuous increase of serum troponin,^100^ decrease of ventricular ejection fraction,^99^ and abnormal electrocardiogram.^100^, ^101^

Hospitalized COVID‐19 patients were associated with a high incidence of cardiac damage, and half of those who recovered with long COVID symptoms had myocardial damage during infection.^102^ The incidence of persistent myocardial injury was variable. A study reported that nearly 60% of recovered patients suffered from persistent (2 months) myocardial inflammation,^99^ while other studies found a small portion of patients still existed cardiac inflammation 2 months after infection, and symptoms relieved or resolved subsequently. Diagnostic tests with different sensitivities and design of studies may contribute to the variable prevalence of cardiac abnormalities across studies. In healthy young people with COVID‐19 infection, the prevalence of cardiac involvement after recovery was low. A retrospective study of 3018 COVID‐19 athletes conducted in 2020 showed that less than 3% of those experienced long‐term cardiac involvement.^103^ Other studies showed similar results,^104^, ^105^ indicating the low prevalence of persistent cardiac involvement in healthy young patients recovered from COVID‐19.

Based on the symptoms of myocardial injury, three major mechanisms in the acute phase of infection are put forward. (1) Direct virus infection of cardiomyocytes and endothelial cells. SARS‐CoV‐2 infects human cardiomyocytes in vitro, with viral particles detected in living human heart slices after infection.^106^ The normal expression of ACE2 is critical for cardiac function, and the injury induced by SARS‐CoV‐2 infection on cardiomyocytes may down‐regulate the membrane ACE2 expression both at mRNA and protein levels, contributing to myocardial abnormality.^107^ (2) Myocardial ischemia caused by microvascular thrombosis. (3) Activation of systemic inflammatory caused by virus infection that indirectly leads to cardiac injury.^108^ RNA fragments of SARS‐CoV‐2 were detected in non‐infectious COVID‐19 convalescents, and RNA fragments as antigens can lead to continuous activation of the body's immunity.^109^

### 2.7 Intestinal involvement

Gastrointestinal (GI) symptoms are the main extra‐pulmonary manifestations of COVID‐19, such as diarrhea, vomiting, and abdominal pain, and the incidence is between 40% and 60%.^110^, ^111^ The prevalence of GI symptoms in female patients was significantly higher than in male patients.^112^ SARS‐CoV‐2 was detected in small intestinal epithelial cells of COVID‐19 patients.^113^ The gut microbiota composition of COVID‐19 patients distinguished from that of healthy and H1N1‐infected individuals.^114^ Some COVID‐19 patients with intestinal symptoms at admission remained symptomatic and unrecovered intestinal phenotype after 1–2 months.^115^ Persistent high levels of phenylacetylglutamine, uric acid, and salsolinol were observed in the fecal of COVID‐19 patients, which are deleterious metabolites derived by microorganisms,^116^ indicating the disturbance of intestinal homeostasis in patients recovered from COVID‐19. The changes in gut microbiota of COVID‐19 patients could last for 6 months after disease resolution, manifested as the reduction of gut microbial diversity and the relative abundance of *bifidobacterium*, a beneficial gut commensal.^117^, ^118^ Severe COVID‐19 patients showed lower microbiota diversity at convalescence.^118^

Although administration of antibiotics may be a treatment regimen for some patients, data suggested that the use of antibiotics did not affect the profile of gut microbial at 6 months post‐infection.^117^ Intestinal SARS‐CoV‐2 infection resulted in elevated levels of IL‐18, an inflammatory cytokine, in the gut of patients, suggesting that alterations in the ecology of gut microbiota might be directly related to SARS‐CoV‐2‐induced inflammatory cytokine production in the gut.^114^ In pediatric COVID‐19 patients with inflammatory multisystem syndrome, intestinal symptoms such as ascites, abdominal pain, intestinal wall thickening, and mesenteric inflammation might occur during the infection period, and the above symptoms gradually relieve to resolve within 2 months after infection.^119^ The lung–gut axis is a bidirectional pathway proposed in recent years, and the two systems interact with each other through immune signaling molecules. Pulmonary involvement often occurs together with chronic GI diseases.^120^ Studies had shown that patients with unrestored gut microbial richness were accompanied by reduced pulmonary function 6 months after SARS‐CoV‐2 infection. Similarly, patients who still had respiratory involvement at 3 months after infection had altered gut microbial profile and decreased diversity.^121^ In addition, patients with GI symptoms had higher rates of smell or taste loss, fatigue, and myalgia.^122^ Acute injury and long‐term ecological changes of intestinal tract during and after COVID‐19 may be caused by direct infection of absorbable intestinal cells in the ileum and colon, which highly express ACE2 receptor, leading to intestinal cell damage and subsequent intestinal microecological change.^112^

### 2.8 Immunological dysfunction

Immunological dysfunction is not a single organ symptom, and the continuous activation after the recovery from COVID‐19 is partly account for the residual symptoms in multiple organs. Immunological dysfunction persisted for 8 months after COVID‐19, which was independent of the disease severity.^123^, ^124^ COVID‐19 convalescents presented significant elevated pro‐inflammatory cytokines such as interferon (IFN)‐β, IFN‐λ1, IL‐6, and IL‐8 than individuals who were healthy or infected with prevalent human coronaviruses, and convalescents with long COVID had highly activated innate immune cells.^123^, ^125^ The profound natural killer T (NKT) cell impairment was observed at 2 months post‐infection, with decrease in NKT‐like cells.^126^ Also, RNA sequencing revealed the significant alternations in gene expression for up to 6 months after COVID‐19.^124^ Persistent changes in the peripheral immune system might affect the immune response of COVID‐19 patients to other infections after recovery, and the persistent activation of immune system might aggravate other chronic diseases.^124^

In addition, the deaths of tuberculosis globally during the COVID‐19 pandemic increased for the first time in a decade, while the tuberculosis patients has decreased, suggesting the increase of the mortality of tuberculosis patients.^127^ The prolonged use of glucocorticoids for post‐pneumonia treatment might re‐active the tuberculosis. Patients who received chemotherapy weeks after the diagnosis of COVID‐19 seemed to have a decrease rate of developing sequelae, compared with patients without tumors.^128^ It was postulated that chemotherapy might reduce the unopposed pro‐inflammatory signal. Interestingly, the risk of long COVID in cancer patients decreased with age, which also suggests that immunosuppression caused by chemotherapy may work together with the senescence of immune system to protect against the pro‐inflammatory signals.

### 2.9 Kidney involvement

Acute kidney injury (AKI) is common in COVID‐19 hospitalized patients in the early epidemic, with 46% of 3993 hospitalized patients invloved.^129^ However, other studies reported the low incidence of AKI as 0.5% and abnormalities in renal function as 3%–7%.^130^, ^131^ The incidence of AKI was positively associated with the severity of infection. The development of AKI was more frequent in severely and critically ill COVID‐19 patients.^132^ Among patients with AKI, 30%–80% of them have recovered their renal function at discharge.^129^, ^133^ Among the COVID‐19 patients with AKI, some patients with SARS‐CoV‐2 infected after kidney transplantation developed renal transplantation dysfunction.^134^ However, COVID‐19 patients without AKI were not guaranteed not to develop abnormal renal indicators at follow‐up.^135^

The glomerular filtration rate of severe COVID‐19 patients decreased significantly at 3 months after discharge.^136^ COVID‐19 patients who developed AKI had a higher risk of decreased glomerular filtration rate over the first 6 months after hospital discharge than patients without COVID‐19.^133^ In the early phase of the epidemic, AKI involvement and heavy proteinuria were even identified in mild COVID‐19 patients.^137^

The cause of AKI may be the crosstalk of injured organs, systemic effects caused by cytokines,^138^ or the direct infection of kidney cells.^139^ SARS‐CoV‐2 can directly infect kidney cells and induce cell damage, and single‐cell RNA sequencing indicated the infected organoids exhibited activated profibrotic signaling pathways.^139^ It was suggested that anti‐SARS‐CoV‐2 drugs treatment may increase kidney burden, and cause kidney damage. Overall, available data suggested that the incidence of kidney involvement in COVID‐19 patients was low, the AKI was generally identified in severe COVID‐19 patients or COVID‐19 patients have experienced kidney transplantation.

### 2.10 Biochemical abnormalities of liver

Liver injury in patients of early COVID‐19 was characterized by temporary elevation of aminotransferases, SARS‐CoV‐2 was identified in the cytoplasm of hepatocytes, and a research showed 41% of COVID‐19 patients had abnormal liver enzyme levels, and 23.5% of mild cases,^140^ while liver injury was usually transient and resolved with infection resolution.^141^ In other studies, the incidence of abnormal liver function ranged from 14% to 69%.^142^ Some patients were observed severe bile duct injury and liver microvascular lesions in convalescence, suggesting that long COVID could manifest as liver injury.^141^, ^142^ Compared with mild COVID‐19 patients, patients with severe COVID‐19 were more likely to experience elevated liver enzymes, which were initially manifested as elevated aminotransferases, followed by cholestasis. Children with COVID‐19 had a similar prevalence of liver injury to adult patients.^143^ After acute infection, the chronic liver abnormality was 3% among COVID‐19 convalescent.^144^ Chronic liver abnormality mainly manifests as inflammation, ectopic fat, hepatomegaly,^145^ and abnormal biochemical indicators. In addition to the direct damage caused by viral infection, such as other organ involvements, the pro‐inflammatory response induced by SARS‐CoV‐2 infection may contribute to liver enzyme abnormality. Abnormal liver functions may also be related to the administration of certain antiviral agents such as lopinavir/ritonavir and corticosteroids.^143^, ^146^ As the virulence of SARS‐CoV‐2 variants declines, the risk of liver injury during acute phase and convalescence may decrease.

### 2.11 Uncommon long COVID symptoms

Ocular involvement like retinal micro‐vasculopathy is an ocular manifestation of COVID‐19 and may evolve to long‐term symptoms in COVID‐19. COVID‐19 patients with severe pneumonia are more susceptible to ocular involvement. Decreased vascular density and enlarged foveal avascular zone of the foveal and parafoveal retinal vascular network could be identified in patients at 6 months post‐infection,^147^, ^148^ indicating the long‐term evolvement in COVID‐19 patients. The meta‐analysis suggested that COVID‐19 increased the risk of retinal hemorrhages by fivefold, and moderate or severe COVID‐19 patients have more retinal vasculature changes than mild COVID‐19 patients. The prevalence of long‐term ocular involvement in COVID‐19 convalescent is unavailable. The mechanism of long‐term involvement of ocular involvement is inconclusive and poorly comprehensive, and is currently evidenced by microvascular endothelial injury. Therefore, it is suggested that the improvement in vascular involvement may be accompanied by improvement of ocular involvement.

COVID‐19 may exert a negative impact on the sexual function of male. Studies have shown that 19% of patients complained about scrotal discomfort after the diagnosis of COVID‐19, while infectious SARS‐CoV‐2 particle was not detected in the semen of COVID‐19 patients who recovered for 1 week or more.^149^, ^150^ Persistent sexual dysfunction caused by COVID‐19 also included difficulty in erectile function, decreased sexual desire, and difficulty in ejaculation.^151^ The sperm quality of COVID‐19 patients might decrease, and it was expected to take 3 months for recovery. Further follow‐up studies are still needed to confirm whether SARS‐CoV‐2 has long‐term effects on male fertility and testicular endocrine function.

## 3 LONG COVID IN PATIENTS WITH BREAKTHROUGH INFECTION AFTER VACCINATION

Vaccination decreases the probability of hospital admission and mortality among patients with COVID‐19.^3^, ^152^, ^153^ Although vaccination mitigates the severity of SARS‐CoV‐2 infection, long COVID occurred in mild and severe infection.^154^ A community‐based study published in the Lancet Infectious Diseases revealed that patients who had symptoms for 28 days or more post‐infection were halved by two vaccine doses, and higher proportion of asymptomatic patients was seen in vaccinated individuals.^155^ Similar conclusions were also reported by Ayoubkhani et al. Results of this observational study in a community population showed that adenovirus and mRNA vaccines reduced the risk of long COVID such as olfactory impairment, fatigue, and headache for more than 12 weeks in patients that vaccinated after infection.^156^ Arnold et al. suggested that vaccination after SARS‐CoV‐2 infection did not exacerbate the symptoms of long COVID that patients already had.^157^ In addition, a study published in Nature showed the risk of long COVID at 6 months post‐infection was lower in patients vaccinated before infection, compared with unvaccinated individuals, but was still higher than in seasonal influenza patients.^158^ However, another study showed no significant reduction in the incidence of long COVID symptoms after 6 months of infection as long as breakthrough infection occurred.^159^ A preprint paper reported nearly half (24/44) of the patients with SARS‐CoV‐2 breakthrough infection reported long COVID symptoms. Whether vaccination can reduce the incidence of long COVID is an ongoing issue, and due to the deficiency of studies, no definitive conclusion has been reached on the effect of vaccination on the risk of long COVID in patients with breakthrough infection.

## 4 POSSIBLE DIFFERENCES IN SYMPTOMS OF LONG COVID BETWEEN DIFFERENT SARS‐COV‐2 MUTANT INFECTIONS

Most existing long COVID studies were based on patients infected in the first year of the pandemic, while few reports included SARS‐CoV‐2 variants cases. A cross‐sectional study compared the prevalence of long COVID in patients with omicron and other strains. The prevalence of long COVID in omicron patients was less than that of the other strains (5.6% vs. 55.6%, *p* = 0.003).^160^ Olfactory and taste impairment are the most specific symptoms of COVID‐19 and also the main symptoms of long COVID, with an incidence of more than 50% before the epidemic of omicron. The incidence of olfactory impairment caused by omicron infection was significantly reduced compared with during the epidemic of the early strains, alpha, and delta, with about 13%–23% of omicron patients experiencing the olfactory impairment,^161^, ^162^, ^163^ and so did the incidence of taste impairment.^164^ The altered cyto‐tropism of omicron may be responsible for its lower incidence of olfactory impairment. A study in a hamster model showed that omicron reduced the infection in olfactory epithelial cells while increased in seven‐ to ten‐fold in sinonasal epithelium.^165^ SARS‐CoV‐2 employs two pathways for cells entry, which are TMPRSS2‐mediated membrane fusion pathway and cathepsin‐mediated endocytosis pathway. Omicron mainly uses the latter pathway to enter cells. The sustentacular cells in the olfactory epithelium highly express TMPRSS2, and omicron may have a low tropism on these cells, thereby reducing the frequency of anosmia.^164^ The experiment based on human‐induced pluripotent stem cells (hiPSC) derived neurons culture, hACE2 transgenic mice and hamster infection model showed that compared with delta and D614G mutant, the neurotropism, neuro‐invasion, and neurovirulence of omicron are decreasing,^50^ which may be explained by the less replication efficiency in the olfactory mucosa of omicron compared with earlier variants.^50^, ^166^ Nevertheless, some studies also declared that the neurological and psychiatric outcomes were similar between the delta and omicron infection.^167^

SARS‐CoV‐2 infection causes liver injury, and current studies are mostly based on early strains and early variants. Deng et al. compared the incidence of liver injury in 77 delta and 88 omicron patients at the early stage of infection and found that there was no significant difference in the incidence of liver injury in patients (19.4% and 13.8%, respectively), and most of the patients recovered to normal during the follow‐up period.^168^ Patients infected with alpha and delta variants reported fewer long COVID dyspnea than those infected with the early strains.^169^ Fewer patients with comorbidities were admitted to hospital for omicron infection than the past, and the proportion of patients with acute respiratory symptoms was lower.^163^, ^170^ A study from Lancet reported that 2501 of 56,003 people (4.5%) with omicron infection experienced long COVID, and 10.8% (4469/41,361) of those with delta infection reported long COVID.^171^ The odds of long COVID in patients infected with both variants were significantly lower than that of the early strains. Due to the short assessment period, reports on the symptoms and incidence of long COVID in omicron patients are less understood. We hypothesize the risk of olfactory, taste impairment, and respiratory symptoms in omicron cases may be significantly lower than early cases, based on the symptoms of omicron and the existing reports of long COVID. However, the incidence of other symptoms was not reported (Table 1).

**TABLE 1:** Differences of major long COVID symptoms among early strains and variants of concern (VOCs)

Symptoms	early strains	VOCs	Research type	Reference
Any symptoms	–	Delta: 10.8% Omicron: 4.5%	Observational research	^171^
	20% (5 weeks follow‐up) 10% (12 weeks follow‐up)	–	Observational research	Statics form the Office of National Statistics（Britain）
	Other strains^a^: 55.6%	Omicron: 5.6%	Observational research (2–3 months follow‐up)	^160^
Dyspnea	29.35%	Alpha: 13.75% Delta: 12.8%	Observational research (6 months follow‐up)	^169^
Smell dysfunction	21%	–	Meta‐analysis	^172^
	1.5%	Alpha: 2.4% Delta: 14.3%	Observational research (6 months follow‐up)	^169^
	Other strains^a^: 16.7%	Omicron: 0%	Observational research (2–3 months follow‐up)	^160^
Fatigue	68.2%	Alpha: 71.5% Delta: 76.35%	Observational research (6 months follow‐up)	^169^
	Other strains^a^: 5.6%	Omicron: 5.6%	Observational research (2–3 months follow‐up)	^160^
Myalgia	24.9%	Alpha: 33.2% Delta: 33.7%	Observational research (6 months follow‐up)	^169^
Diarrhea	7.4%	Alpha: 5.2% Delta: 15.0%	Observational research (6 months follow‐up)	^169^
Brain fog	10.4%	Alpha: 10.4% Delta: 10.9%	Observational research (6 months follow‐up)	^169^

*Note*: Data were extracted from observational researches that compared long‐term symptoms after COVID among different variants or meta‐analysis on long COVID.

^a^ Morioka et al. compared the prevalence of post‐COVID‐19 condition in patients with omicron infection and patients with other strains infection.

## 5 POTENTIAL THERAPEUTIC INTERVENTIONS OF LONG COVID

Potential therapeutic strategies of long COVID are discussed separately according to symptoms in different organs. Pulmonary impairments appear mildly in relatively healthy population. Mild to moderate COVID‐19 patients with long COVID pulmonary symptoms may consider attending the outpatient pulmonary rehabilitation.^173^ Patients with severe and comorbid conditions are more likely to have persistent pulmonary symptoms. The drugs treatment of persistent pulmonary impairments is mainly for the latter population. Nintedanib and pirfenidone are drugs approved for treating pulmonary fibrosis.^4^ The release of histamine could mediate cytokine storm, and the blockade of histamine receptor had been identified to reduce pulmonary symptoms in COVID‐19 patients,^174^ which suggests that antihistamine treatment can be used to relieve pulmonary symptoms caused by long COVID lung inflammation.

Mitochondrion is a major producer of ROS in cells, which makes it susceptible to the damage raised by oxidative stress. Antioxidants treatment that removes damaging free radicals to alleviate oxidative stress and improve mitochondrial function is under investigation. Antioxidants such as vitamin E, vitamin C, and CoQ10 scavenge free radicals, and are considered as potential approaches to improve chronic fatigue.^175^, ^176^ Mitochondrial‐targeted ubiquinone (MitoQ), which alleviated viral infection through suppressing ROS production in mouse model, is a potential treatment for long COVID neurological involvements.^177^ An observational study gave a preliminarily evidence that nutritional supplement based on antioxidant substances reduce chronic fatigue of COVID‐19 patients at convalescence.^178^

The decrease level of IL‐6 was correlated to the recovery of olfactory impairment.^179^ IL‐6 is a key component in cytokine storm and involves in regulating olfactory neuronal activity. The high expression of IL‐6 is related to severe clinical manifestations.^65^, ^179^ Convalescents plasma (CP) from recovered COVID‐19 patients can be used for the treatment of COVID‐19. Zheng et al. evaluated the prevention effect of CP on the olfactory loss induced by SARS‐CoV‐2 infection in K18‐hACE2 mouse, and indicated that the CP pretreatment did not prevent the loss of smell.^64^ Smell treating, drugs treatment that reduce inflammation are now being explored to improved smell impairment.^180^ Moreover, administration of oral steroids, alpha‐lipoic acid, sodium citrate, omega 3 fatty acids, and intranasal vitamin A may be beneficial for the improvement of olfactory function, although these strategies are preliminary and some are not evaluated in COVID‐19 patients.^181^ Currently, as most of the COVID‐19 patients are infected with omicron, the risk and degree of smell impairment may decrease, and smell impairment may resolve spontaneously without targeted treatment.

The CV system damage caused by COVID‐19 may further damage the heart due to microvascular obstruction,^182^ or develop to multiple abnormal cerebral embolisms or ischemic stroke.^183^ Therefore, monitoring and intervention of CV injury may reduce the long COVID. Biomarkers including C‐reactive protein, ferritin, D‐dimer, and procalcitonin may help to determine thrombotic and vascular long COVID.^184^, ^185^ Anticoagulants are commonly used to prevent and treat venous or arterial thromboembolism, such as heparin. In addition to its anticoagulant effect, heparin has additional anti‐inflammatory potential and may affect the clinical evolution of COVID‐19 patients.^186^ A study found that COVID‐19 patients who received heparin for thromboprophylaxis rarely developed significant disseminated intravascular coagulation.^186^, ^187^ Moreover, the result of case reports has shown that the development of endothelial injury and aberrant immune response was associated with histamine release. Administration of antihistamines was reported to alleviate long COVID symptoms. Histamine antagonist intervention may provide treatment for patients experiencing vascular sequelae.

Treatment of muscle pain sequelae is unclear. Appropriate protein uptake to treat against muscle protein catabolism is a potential therapy for muscle mass restoration.^34^ Supplement with antioxidant substances that inhibit ROS production may be beneficial for recovering the oxidation–reduction homeostasis after SARS‐CoV‐2 infection. Apart from pharmacological intervention, aerobic and resistance training to reduce fatigue and increase strength may be good for skeletal muscle, bone, joint, connective tissue, and cardiorespiratory health of COVID‐19 patients.^87^, ^91^

Reports on potential therapies for post‐acute cardiac abnormalities are lacking. The management of chronic cardiac abnormalities could be performed according to official guidelines from different countries. ACE inhibitors, beta‐receptor blockers, calcium channel blockers, and Ang receptor blockers, which are used as antihypertensive drugs and protect the CV system,^188^ are recommended for treating COVID‐19 convalescent with acute and chronic CV abnormalities.^189^ ACE2 activator was shown to improve cardiac function by suppressing renal and cardiac fibrosis in spontaneous hypertension.^189^ Other therapies, such as anti‐inflammatory therapy and RAAS therapy, are under clinical trials.^190^ Apart from pharmacological interventions, COVID‐19 convalescent with asymptomatic cardiac abnormalities, who require less pharmacological intervention, are advised to avoid intense exercise at convalescence.^191^

Clinical trials have documented that probiotic and prebiotic have the potential to construct intestinal environment,^192^ and may help to improve the acute and chronic intestinal symptoms.^193^ Dietary fibers are ideal carbon source of intestinal microbes, the increased intake of dietary fibers may regulate and improve microbiota diversity. In addition, supplement with anti‐inflammatory bacterial species is considered to accelerate the restoration of gut microbiota after COVID‐19^199^.

Based on the serological manifestation of patients experiencing immunological dysfunction, suggested treatment of immunological dysfunction focuses on immunoregulation. Blockade of pro‐inflammatory cytokine such as IL‐6, TNF, and IFN is proposed to suppress the over‐activated immune response and improve immune dysregulation. Moreover, blockade of histamine receptor may play a key role in reducing inflammation.

As chronic kidney involvement does not commonly exist in COVID‐19 patients, recommended management is still poorly investigated and developed. Based on the mechanism of kidney damage, putative treatment for post‐acute kidney damage may include alleviating the inflammatory factor storms. Administration of immunosuppressive drugs, pro‐inflammatory cytokines inhibitors, and immune signaling pathways regulators may be helpful for improving COVID‐19 associated kidney involvement.^194^

Current guidance for long‐term liver abnormality caused by COVID‐19 is unavailable. However, the WHO has issued the guidance for liver disease patients with COVID‐19.^195^

Table 2 summarizes the potential mechanisms and suggested treatment of long COVID.

**TABLE 2:** Putative mechanism and suggested treatment of long COVID in multiple systems

Involvement	Putative mechanism	Suggested treatment
Pulmonary and respiratory involvement	Pulmonary fibrosis Severe cytokine storm	Antifibrotic drug Administration of antihistamines reagent
Neurological involvement	Brain hypoxemia Neuronal necrosis Mitochondrial dysfunction Neurotoxicity caused by inflammatory responses and cytokine release	Antidepressants Pain medications Mitochondrial modulator: MitoQ Free radicals scavenger: vitamin C, vitamin E, and CoQ10 Immunomodulatory therapy^196^
Olfactory involvement	Viral infection on sustentacular cells Cytokines released by vascular injury Down‐regulation of genes related to olfactory receptor	Smell treatment Anti‐inflammation treatment Drug therapy: oral steroids, alpha‐lipoic acid, sodium citrate, omega 3 fatty acids, and intranasal vitamin A
Vascular involvement	Viral infection on perivascular cardiomyocytes and pericytes High pro‐inflammatory cytokine response	Administration of antihistamines reagent^197^ Administration of anticoagulant: heparin Immunomodulatory therapy and blockade of pro‐inflammatory cytokines
Muscular involvement	Systemic inflammation Muscle fibrosis and degradation caused by the dysfunction of ACE2–Ang 1–7–AT1 axis Low partial pressure of oxygen in venous blood	Increased protein intake Supplement with antioxidant substance Aerobic and resistance training
Cardiac involvement	Direct virus infection of cardiomyocytes and endothelial cells Myocardial ischemia caused by microvascular thrombosis Activation of systemic inflammatory	Anti‐arrhythmic drugs ACE inhibitors, beta blockers, calcium channel blockers, and angiotensin receptor blockers Cardiac anti‐inflammatory therapy: colchicine Renin–angiotensin–aldosterone system inhibition Avoid intense exercise
Intestinal involvement	Viral infection on absorbable intestinal cells Inflammation Alternation of gut microbiota	Supplementation of anti‐inflammatory bacterial species Uptake of prebiotic and probiotic Dietary therapy
Immunological dysfunction	Over expression of pro‐inflammatory cytokines Highly activated innate immune cell	Immunomodulatory therapy and blockade of pro‐inflammatory cytokines Administration of antihistamines reagent
Kidney involvement	Viral direct infection with renal cells Crosstalk of injured organs High pro‐inflammatory cytokine response	Immunomodulatory therapy and blockade of pro‐inflammatory cytokines
Liver involvement	Viral infection on hepatocytes Activation of systemic inflammatory	The guidance for liver disease patients with COVID‐19 issued by WHO

Abbreviations: Ang, angiotensin; WHO, World Health Organization.

## 6 DISCUSSION

The early strains of SARS‐CoV‐2 and variants before omicron mainly infect lungs, and cells in the heart, kidney, intestine, liver, and other systems.^170^ It is widely recognized that after the acute infection, a substantial proportion of convalescents suffer from long‐term symptoms for several weeks to 2 years, designated as long COVID. According to a large number of follow‐up studies, COVID‐19 convalescents present one or more long COVID symptoms, with the incidence ranging from 0.9% to 80% in different periods of the epidemic and different assessment period.^171^, ^198^, ^199^ At present, two main mechanisms account for the pathogenesis of long COVID: (1) SARS‐CoV‐2 directly infects cells that express functional receptor ACE2 results in the damage to organs and systems, and contributes to the persistent injury. ACE2 is highly expressed in cardiac, kidney, lung, and vascular cells; (2) SARS‐CoV‐2 infection leads to persistent inflammatory response, manifested by the dysfunction of immune cells such as T cells and the continuous elevation of pro‐inflammatory factors, such as IL‐6 and TNF.^123^ The persistent existence of SARS‐CoV‐2 antigens such as the receptor binding domain (RBD) of Spike, RdRp, and nucleocapsid antigen (not infectious virus) enables a continuously activated and robust immune response, which contributes to the development of long COVID.^109^, ^200^ Recently, researches found alterations in genes express in olfactory epithelium and involve in the metabolism of odor molecules, indicating a new mechanism of long COVID smell dysfunction.

Most COVID‐19 convalescents with persistent SARS‐CoV‐2 antigens reported post‐infection symptoms, while patients without viral antigens did not.^109^ Female has a risk factor for most post‐infection symptoms, but is lack of interpretation. The severity of the disease, the number of symptoms in the acute infection phase, and the presence of comorbidities are positively associated with the severity of long COVID.^151^, ^201^ In addition, the burden of long COVID is associated with the age of patients, with the elderly patients exhibit a higher proportion of long COVID.^26^ A study showed that 51.5% of the elderly patients reported one or more sequelae including fatigue, sweating, chest tightness, muscle soreness, cough, anxiety, palpitations, lower limb edema, taste changes, hyposmia, and so on.^202^ However, the pediatric and teenage patients showed milder post‐infection symptoms and lower incidence as 0.5%–1%,^203^ due to the lower predisposition to pro‐inflammatory states and less comorbidities.

**FIGURE 4:**
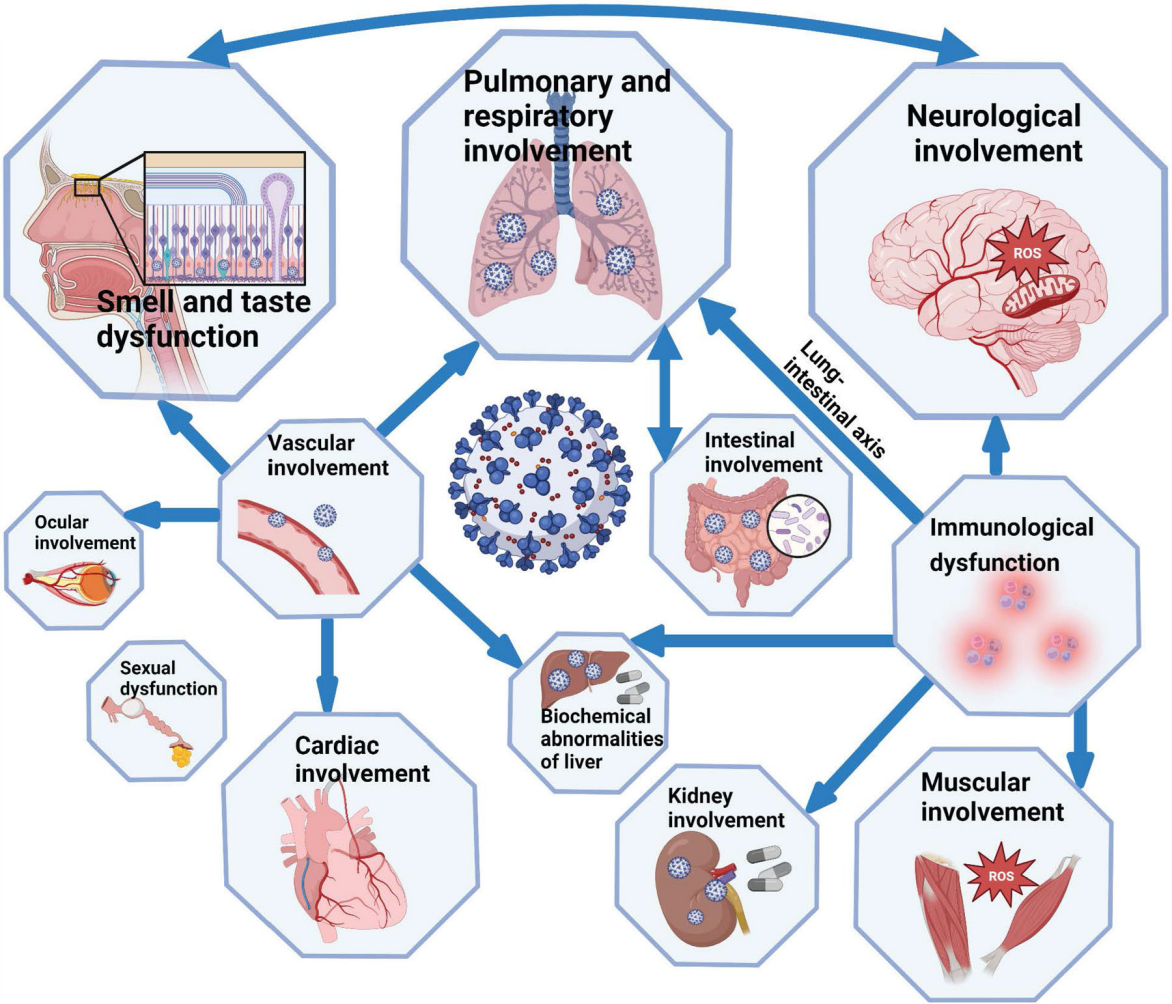
Long COVID symptoms discussed in the review. Correlations between different symptoms are showed by arrows. The relative organs size in the diagram roughly indicate their prevalence in long COVID population

In addition, manifestations of long COVID in different organs and systems are correlated (Figure 4). The lung–intestinal axis is a bidirectional pathway proposed in recent years. These two systems interact with each other through immune signaling molecules. Vascular damage may cause injury in organs including heart, eyes, and liver. Patients with GI symptoms had higher rates of the loss of smell or taste, fatigue, and myalgia. Abnormalities in the nervous system can lead to impairment of smell and taste, and viral infection of olfactory epithelium cells and neurons may further impair the central nervous system. Involvement of kidney, muscle, nervous system, and liver may be caused by the pro‐inflammatory condition. In addition to the symptoms summarized, there are also some less reported symptoms, such as alopecia and decreased libido.^151^ SARS‐CoV‐2 has continuously mutated since the beginning of the pandemic, with a general trend toward increased infectivity and reduced severity.

The rate of long COVID continuously changes with different variants.^204^ A latest study shows that the risk of long COVID in omicron cases was the half of who infected with delta,^171^ and the most notable, the incidence of smell dysfunction in omicron cases was significantly reduced compared with the early strains and other mutants.^161^, ^162^, ^163^, ^164^ The reduced risk of long COVID in omicron and delta infections compared with the early strains may attribute to the diminished virulence of SARS‐CoV‐2 and the widespread of vaccination. The positive effect of vaccination on reducing the risk of long COVID is inconclusive, although some studies have shown that vaccination reduced the risk for several post‐infection symptoms.^159^ However, due to the increasing cases of omicron infection, the population of convalescent with long COVID should not be ignored. Based on our review, current research on the treatment of long COVID and long COVID associated with omicron is insufficient. Therefore, future studies are expected to focus on the management and dynamic change of long COVID symptoms. The research and treatments on long COVID are needed to be updated to support the public health systems.

## AUTHOR CONTRIBUTIONS

H.F. and Y.T. designed the research. S.H., K.W., Z.C., M.H., R.H., N.F., L.S., and Q.L. read and analyzed the references and drafted the manuscript. S.H., H.F., and Y.T. revised the manuscript. All authors have read and approved the article.

## CONFLICT OF INTEREST

The authors declare that they have no conflicts of interest.

## ETHICS STATEMENT

Not applicable.

## Data Availability

The data included in this study are available upon request from the corresponding author.
